# mTOR signaling and Alzheimer's disease: What we know and where we are?

**DOI:** 10.1111/cns.14463

**Published:** 2023-09-18

**Authors:** Samin Davoody, Afsaneh Asgari Taei, Pariya Khodabakhsh, Leila Dargahi

**Affiliations:** ^1^ Student Research Committee, School of Medicine Shahid Beheshti University of Medical Sciences Tehran Iran; ^2^ Neuroscience Research Center Shahid Beheshti University of Medical Sciences Tehran Iran; ^3^ Department of Neurophysiology Institute of Physiology, Eberhard Karls University of Tübingen Tübingen Germany; ^4^ Neurobiology Research Center Shahid Beheshti University of Medical Sciences Tehran Iran

**Keywords:** Alzheimer's disease, mammalian target of rapamycin, pathogenesis, rapamycin

## Abstract

Despite the great body of research done on Alzheimer's disease, the underlying mechanisms have not been vividly investigated. To date, the accumulation of amyloid‐beta plaques and tau tangles constitutes the hallmark of the disease; however, dysregulation of the mammalian target of rapamycin (mTOR) seems to be significantly involved in the pathogenesis of the disease as well. mTOR, as a serine–threonine protein kinase, was previously known for controlling many cellular functions such as cell size, autophagy, and metabolism. In this regard, mammalian target of rapamycin complex 1 (mTORC1) may leave anti‐aging impacts by robustly inhibiting autophagy, a mechanism that inhibits the accumulation of damaged protein aggregate and dysfunctional organelles. Formation and aggregation of neurofibrillary tangles and amyloid‐beta plaques seem to be significantly regulated by mTOR signaling. Understanding the underlying mechanisms and connection between mTOR signaling and AD may suggest conducting clinical trials assessing the efficacy of rapamycin, as an mTOR inhibitor drug, in managing AD or may help develop other medications. In this literature review, we aim to elaborate mTOR signaling network mainly in the brain, point to gaps of knowledge, and define how and in which ways mTOR signaling can be connected with AD pathogenesis and symptoms.

## Graphical abstract

mTOR signaling and Alzheimer's disease (AD). Upregulation of mTOR activity appears to be involved in the main pathological cascade of AD, and mTOR inhibition or targeting its downstream substrates could supply attractive avenues for AD treatment.

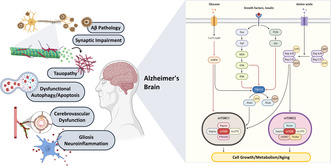

## 1 INTRODUCTION

Dementia is a clinical syndrome characterized by a progressive decline in various cognitive domains which disrupt independent functions.^1^ The World Alzheimer Report 2019 claims that approximately 50 million running cases of dementia exist in the world and will almost be threefold by 2050.^2^ Alzheimer's disease (AD), the most common cause of dementia, is becoming a global age‐related health issue.^3^ The World Health Organization predicts that by 2040, neurodegenerative diseases such as AD and other causes of dementia will outdo cancer in death rates and become the second leading cause of death in the world.^4^ Regarding the dramatic growth of the older population, it is expected that 8%–10% of the older people in Iran will be affected by AD over the next 2–3 decades.^5^ In addition to the increasing average age, as an important public health concern in many communities, high care costs for AD highlight the need to discover efficient treatment approaches for AD. To this end, unraveling the underlying molecular and cellular mechanisms involved in the pathogenesis and prognosis of AD appears essential. Despite the various valuable research conducted so far on investigating the pathogenesis of AD, there are still many gaps to be filled and questions to be answered.

More than 95% of cases of AD occur sporadically, without any familial history. It is important to mention that familial forms of AD result from specific gene mutations in amyloid precursor protein (APP), presenilin 1 (PS1), PS2, and apolipoprotein E (APOE).^6^ AD is characterized by the presence of extracellular amyloid β (Aβ) plaques, the accumulation of hyper‐phosphorylated tau in the form of intracellular neurofibrillary tangles (NFTs), neural cell death, and loss of synaptic connections.^7^ Furthermore, scientific literature suggests that dysregulated cell survival and death mechanisms may also contribute to the development of AD. For example, disturbances in mammalian/mechanistic target of rapamycin (mTOR) signaling pathways could initiate a series of events that lead to AD pathogenesis.^8^

mTOR interacts with several molecules and is contained within at least two major specific complexes named the mTOR complex 1 (mTORC1) and the mTOR complex 2 (mTORC2). Today, the medical scientific community is experiencing an explosion in the information generated on the role of mTOR. Although a lot has been learned about the physiological involvement of mTORC1, we are still way behind and in the early stages of discovering the role and participation of mTORC2 in various diseases. It would be interesting to learn how a soil sample leading to the discovery of a drug named rapamycin led to the whole discovery of mTOR. In this regard, rapamycin was first discovered in 1964 in a soil sample from the island of Rapa Nui (Easter Island). Sehgal and his colleagues isolated a new macrolide with potent antifungal properties from a Streptomyces hygroscopicus soil bacterium, which they named “rapamycin.”^9^ In 1984, Eng et al.^10^ discovered the inhibitory effect of rapamycin on tumor growth at any stage. Years later, the immunosuppressant and chemotherapeutic properties of rapamycin became FDA‐approved.^11^

To the best of available knowledge, rapamycin, as an mTOR inhibitor drug, inhibits mTORC1. Although mTORC2 is insensitive to acute rapamycin treatment, some evidence proposes that chronic treatment with rapamycin can inhibit mTORC2 as well as mTORC1 and its consequential stream.^12^ Animal studies suggest that mTOR inhibitor drugs, such as rapamycin, may aid to lower the AD progression or ameliorate cognitive impairments of AD^12^, ^13^; however, based on the data retrieved from clinicaltrial.gov, there is no terminated clinical trial on elucidating the effects of rapamycin on delaying the progression of AD to date. A clinical trial entitled “Cognition, Age, and RaPamycin Effectiveness—Downregulation of the mTOR Pathway” NCT number: NCT04200911 is in the active stage. Additionally, a clinical trial entitled “Rapamycin—Effects on Alzheimer's and Cognitive Health” NCT number: NCT04200911 is in the recruiting stage. In all, the specific role of mTOR in AD onset and pathogenesis, and the role of rapamycin, in delaying the progression of AD remain elusive and much work remains to be done.

The present review focuses on mTOR signaling network, its role in normal aging, and underlying mechanisms involved in AD pathology. We also summarized preclinical studies addressing therapeutic potentials of mTOR network targeting in animal models of AD. Finally, we discussed the clinical prospects regarding the translation of mTOR targeting.

## 2 mTOR AND BRAIN PHYSIOLOGY

mTOR, a serine–threonine protein kinase, plays a crucial role in regulating various fundamental cellular functions in both neural and non‐neural cells. These functions encompass cell size, mitochondria, protein synthesis, autophagy, energy metabolism, lysosome biogenesis, and lipid metabolism.^14^ The roles of mTOR are achieved by merging extracellular instructions with information on metabolic resources; therefore, mTOR signaling controls the rate of catabolism and anabolism. Generally, mTORC1 controls growth, autophagy, and protein synthesis, and mTORC2 is involved in apoptosis,^15^ osmotic homeostasis maintenance,^16^ somatic and cancer cells' survival, and cytoskeletal organization.^11^ Furthermore, specific functions of mTOR in the brain include axonal regeneration and spouting, myelination, and channel expression. Moreover, nutrients and neurotransmitters that suppress autophagy and enhance protein synthesis and neurotrophic factors may activate mTOR signaling pathways. Since these processes have a role in neural growth by promoting neural elongation and branching, synaptic plasticity, and neural differentiation; therefore, dysregulated mTOR signaling may impair neural regeneration and development. It can be expected that dysfunctional mTOR pathway is associated with neurological disorders. In particular, inhibiting mTOR signaling may bring about neurodegeneration. On the other hand, mTOR signaling hyperactivation may damage the development of neurons and glia and lead to brain malformation.^17^ In this context, inhibition of mTORC1 can be useful in treating some acute neurological conditions including epilepsy, brain tumors, and cognitive impairment.^14^

Studies have demonstrated that increasing mTORC1 signaling by upregulating a constitutively active form of Akt or removing PTEN, a negative regulator of the Akt/mTOR pathway, can have neuroprotective effects.^18^, ^19^ These effects may be attributed to the reduction in endoplasmic reticulum (ER) stress, decreased production of reactive oxygen species (ROS), enhanced mitochondrial biogenesis, and elevated energy metabolism stimulated by mTORC1.^14^ By promoting these beneficial processes, mTORC1 activation can potentially safeguard neurons and contribute to their overall well‐being.^14^ In various models of central nervous system (CNS) injury, the PTEN/AKT/mTOR pathway tends to become inactive. However, functional recovery in injured neurons, such as through axonal sprouting and regeneration, can be achieved by upregulating mTOR signaling.^18^ Furthermore, Akt/mTOR signaling has been associated with neurogenesis. Recent research demonstrated the expression of Akt3 protein in neuroblasts, mature newborn neurons, and progenitor cells. The study revealed that Akt3‐mTOR signaling was closely linked to neurite growth, proliferation of neural progenitor cells, and neurite outgrowth in the hippocampal dentate gyrus.^20^ Moreover, mTORC1 activity is closely linked to CNS myelination, whereas mTORC2 plays a negligible role in this process. Maintaining balanced mTORC1 activity in oligodendrocytes is crucial for efficient CNS myelination.^21^ Genetic studies have shown that Rheb1, through activating mTORC1, plays a vital role in myelination and the differentiation of oligodendrocyte precursor cells (OPCs).^22^ However, hyper‐activated mTOR signaling may be detrimental in proteinopathies where an increased activity level of autophagy is needed to suppress the pathogenesis.^14^ It is important to note that the functions of mTOR signaling in the brain extend beyond synaptic plasticity, circadian rhythm, memory, and cognition.^14^ The mTOR complexes, their signal transduction pathways, and their various functions are summarized in the subsequent sections of this review.

### 2.1 mTOR complex

mTOR belongs to the phosphatidylinositol‐3 kinases (PI3K)‐related kinase (PIKK) family and is a dual‐specificity protein kinase that can phosphorylate serine, threonine, and tyrosine residues. mTOR constitutes several complexes including mTORC1 and mTORC2. Common components of mTORC1 and mTORC2 include mTOR itself, the target of rapamycin complex subunit LST8 (mLST8 also known as GβL), and DEP domain‐containing protein 6 (Deptor). Specific components of mTORC1 are listed as the regulatory protein associated with mTOR (Raptor) and the 40 kDa proline‐rich Akt substrate (PRAS40). Alternatively, the unique components within mTORC2 include rapamycin‐insensitive companion of mammalian target of rapamycin (Rictor), mammalian stress‐activated protein kinase‐interacting protein 1 (MAPKAP1 or mSIN1), and protein observed with Rictor‐1 (Protor‐1).^23^ All the main components of mTORC and their functions are summarized in Table 1. The recent evidence suggests that mTOR can be found in at least two other complexes. The recent research focuses on a putative novel mTOR complex involving G‐protein‐coupled receptor kinase‐interacting protein 1 (GIT1) lacking Raptor or Rictor, which is identified in mouse astrocytes and is crucial for mTOR‐mediated astrocyte survival.^24^ Furthermore, another complex named mTORC3 is present in many cancer cell types which mTORC3 lacks Raptor, Rictor, mSIN1, and mSLT8; however, E26 transformation specific (ETS) translocation variant 7 (ETV7) transcription factor and mTOR seem to be the core components within mTORC3. Appealingly, despite the inhibitory effect of mTOR kinase inhibitors (TORKIs) on mTORC3, the in vitro kinase activity of mTORC3 seems to be resistant to rapamycin.^25^

**TABLE 1:** The main mTOR complexes components and their functions.

mTORC component	Function
mTOR	*Kinase*: serine–threonine protein kinase
mLST8	*Positive regulator*: acts as positive regulator to promote the mTORC1 signaling
Deptor	*Negative regulator*: suppresses mTOR activity and negatively regulates both mTORC1 and mTORC2
Raptor	*Substrate recognizing component*: Scaffold protein determining mTORC1 specific substrate regulates the assembly and the localization, promotes the mTORC1 activity, essential for rapamycin sensitivity
PRAS40	*Negative regulator*: As both component and substrate of mTORC1, suppressing TORC1 activity through its association with Raptor, while its phosphorylation by mTORC1 enhances mTORC1 signaling
Rictor	*Substrate recognizing component*: Scaffold protein determining mTORC2 specific substrate and regulates the assembly, promotes the mTORC2 activity
mSIN1	*Positive regulator*: Scaffold protein regulating the assembly, essential for the integrity of mTORC2, necessary for mTORC2 to activate Akt
Protor‐1	*Positive regulator*: It may play a role in enabling mTORC2 to efficiently activate SGK‐1

### 2.2 The mTOR signaling

The mTORC1 activation is regulated by different signals such as growth factors, insulin, amino acids, nutrients, energy status, and cellular stressors including hypoxia, osmotic stress, and oxidative stress.^26^ The upstream components of the signaling pathways of mTOR as well as the downstream targets have been explained below.

#### 2.2.1 Upstream signaling

The mTORC1 signaling pathway in the brain is triggered through extracellular activators including insulin, insulin‐like growth factor 1 (IGF1), brain‐derived neurotrophic factor (BDNF), vascular endothelial growth factor (VEGF), ciliary neurotrophic factor (CNTF), glutamate, and guidance molecules that inhibit center upstream regulator of mTORC1 signaling called tuberous sclerosis complex (TSC).^17^, ^27^ TSC is a heterotrimeric complex comprising TSC1 (hamartin), TSC2 (tuberin), and TBC1D7 which acts as a GTPase‐activating protein (GAP) to inhibit Ras homolog enriched in the brain (Rheb). To illustrate, Rheb is a small GTPase that binds to mTORC1 on the surface of lysosomes and activates it.^28^ Although there is a functional GAP domain in the TSC2 protein, TSC1 is required to stabilize TSC2 and both are involved in the functionality of this heterodimer.^27^

Activation of growth factors by binding to tyrosine kinase receptors leads to induction of Akt‐dependent phosphorylation of TSC2. This multi‐site phosphorylation inhibits TSC1/TSC2 complex by dissociating it from the lysosomal membrane. In addition to PI3K‐Akt, multiple upstream signaling pathways, including extracellular‐regulated kinase (ERK), ribosomal S6 kinase (p90‐RSK), and glycogen synthase kinase 3β (GSK‐3β), promote mTORC1 activity through phosphorylation of GAP and consequently suppression of TSC2.^15^ In neurons, G‐protein coupled receptors (GPCRs), including metabotropic glutamate μ‐opioid and cannabinoid receptors, transduce signals to Akt and/or MAPK and stimulate mTORC1 activation.^17^ The mTORC1 responds to intracellular stressors such as inflammation, hypoxia, low level of ATP, and DNA damage which can activate the TSC complex to suppress the mTORC1 pathway.^26^

The mTORC1 network is well described, but mTORC2 signaling is relatively poorly defined. The mTORC2 is primarily stimulated by insulin/PI3K signaling. Although growth factors trigger mTORC2 activation by the PI3K pathway, energy status and amino acids are not able to stimulate the mTORC2 sufficiently.^28^, ^29^ It has been shown that mTORC2 phosphorylation by Akt is accomplished on the surface of the endoplasmic reticulum,^30^ and insulin‐PI3K signaling stimulates mTORC2‐ribosome association.^31^ While TSC1/TSC2 is a vital negative regulator of mTORC1, this complex may promote the mTORC2 activity in a manner independent of its GAP activity toward Rheb.^32^ Furthermore, there is a negative feedback regulation of insulin/PI3K and mTORC2 signaling by mTORC1 which brings about the control of mTORC2 network by mTORC1.^33^

#### 2.2.2 Downstream signaling

The mTORC1 regulates protein synthesis through phosphorylation of the eukaryotic initiation factor 4E‐binding protein 1 (4E‐BP1) and the p70 ribosomal S6 kinase 1 (S6K1). The phosphorylation of S6K1 by mTORC1 and activation of several substrates through S6K1 promote the initiation of mRNA translation, elongation, and also ribosome biogenesis. The phosphorylation of 4E‐BP1 by mTORC1 causes dissociation of 4E‐BP1 from eIF‐4E, binding eIF‐4E to eIF‐4G, and finally promoting cap‐dependent translation.^15^

The mTORC1 impacts autophagosome biogenesis through phosphorylation of unc‐51‐like kinase 1 (ULK1) and its interacting proteins including the autophagy‐related protein 13 (ATG13) and the focal adhesion kinase family interacting protein of 200 kDa (FIP200) which in turn leads to suppression of autophagy process.^34^ The mTORC1 activates sterol‐responsive element binding protein‐1,2 (SREBP‐1,2) and peroxisome proliferator‐activated receptor‐γ (PPAR‐γ), two transcription factors which regulate the expression of metabolic genes encoding proteins involved in de novo lipid synthesis. Besides controlling the protein and lipid synthesis by mTORC1, mTORC1 affects ribosome biogenesis and anabolic growth by promoting the production of required nucleotides for RNA and DNA synthesis.^35^

The mRTC2 impacts cell survival and proliferation through phosphorylating AGC (PKA/PKG/PKC) family of protein kinases and serum‐ and glucocorticoid‐induced kinase 1 (SGK‐1). This function is mainly achieved by Akt phosphorylation and causing its maximal activation.^36^ Akt prevents the pro‐apoptotic characteristics of Forkhead box protein O1 (FOXO1) and FOXO3 by phosphorylating them.^28^ PKCα, PKCδ, and PKCζ, as several members of the PKC family, are substrates of mTORC2 which contribute to different aspects of the regulation of cytoskeletal organization and cell migration.^27^ Furthermore, mTORC2 phosphorylates and activates SGK1, a molecule involved in the regulation of neuronal functions, and has inhibitory effects on FOXO1/3 activities.^37^

## 3 mTOR IN NORMAL AGING

It is evident that the functions of mTOR exceed proliferation and the signaling participates in coordinating the cell‐tailored metabolic programs to regulate cell growth and various biological processes such as cellular senescence, lifespan, and aging. To the present date, rapamycin, as an mTOR inhibitor pharmacological substance, is the substance that can postpone aging in all model organisms tested, as well as the only one in mammals.^38^ The first evidence that mTOR is a cellular lifespan regulator comes from the study on the fruit fly Drosophila melanogaster^39^ and nematode Caenorhabditis elegans.^40^ Later, in 2009, Harrison et al.^41^ suggested that when rapamycin, as an mTOR inhibitor drug, is administered to genetically heterogeneous mice from 600 days of age (relatively equivalent to 60 years of age in humans), it can prolong the median and maximal longevity. Considering age with 90% mortality, administration of rapamycin led to elevated lifespan: about 14% in female mice and 9% in males. A great body of further studies confirmed that inhibition of mTOR by specific chemicals can prolong life^42^; however, the impact of mTOR inhibitor drug on longevity was indicated dose‐dependent and not as robust as expected.^43^ Animal studies indicated that deletion of S6K1 protects against age‐related pathologies and increases lifespan.^44^ The life‐increasing effects of mTOR inhibitor drugs may be related to the inhibition of mTORC1, and the inhibition of mTORC2 may even leave dire effects on the inhibitory effect of insulin‐mediated hepatic gluconeogenesis which is controlled by mTORC2.^45^, ^46^

It is now well‐accepted that mTOR inhibition can delay age‐related diseases. However, the putative anti‐aging effects of mTOR inhibitor drugs and the underlying mechanisms are yet to be investigated.

It is now widely acknowledged that mTOR inhibition has the potential to delay age‐related diseases, but the specific anti‐aging effects of mTOR inhibitor drugs and their underlying mechanisms still require further investigation.^38^ Aging is characterized by several hallmarks, including genome instability, accumulation of misfolded proteins, telomere shortening, epigenetic changes, disruption of nutrient‐sensing pathways, cellular senescence, altered intracellular communication, stem cell exhaustion, and impaired mitochondrial function.^47^ mTORC1 is known for regulating mRNA translation by phosphorylating 4E‐BP1 and 4E‐BP2, and ribosomal activity by phosphorylating S6K1 and S6K2, suggesting that the overall decline in mRNA translation caused by mTORC1 inhibition delays aging.^38^, ^48^ Furthermore, mTORC1 has a strong inhibitory effect on autophagy, a cellular mechanism that helps prevent the accumulation of damaged proteins and dysfunctional organelles, thereby exerting anti‐aging effects.^49^ Recent research has also indicated that the mTORC1/p70S6K pathway interacts with stress‐induced senescence of mesenchymal stem cells (MSCs), which contributes to age‐related tissue dysfunction.^50^ Moreover, chronic mTOR inhibition by rapamycin could increase chaperones levels which in turn maintain proteostasis, while its failure leads to aging as well as even AD development.^51^ Although significant amount of animal studies have confirmed that mTOR inhibitor drugs can prolong lifespan; yet, the underlying mechanisms and the actual lifespan‐enhancing effects of mTOR inhibitor drugs need to be further studied. If the aging can be postponed by mTOR inhibitor drugs more specifically, then the putative effects of these drugs on AD can be more easily elucidated.

## 4 mTOR AND AD PATHOLOGY

mTOR signaling plays a significant role in the pathogenic processes of AD, and its underlying mechanisms are discussed below. The amyloid hypothesis is dominating AD pathogenesis and guiding treatment development. In this regard, the imbalanced Aβ production and clearance seem to initiate cascades that result in increasing Aβ levels, and consequent oligomer and plaque aggregation. The failure of synapses and the inflammation they trigger lead to altered neuronal ionic homeostasis, tau hyperphosphorylation, oxidative injury, and intracellular neurofibrillary tangle formation^52^ Although available literature suggests that Aβ deposition is occurred many years before when the AD symptoms are manifested,^53^, ^54^ researchers have not been able yet to clearly define a chronological cascade in pathologic events of AD due to multiple uninvestigated interactions^55^, ^56^; however, piled‐up toxic proteins and protein misfolding are mainly considered as causative events resulting in neuronal loss and neurodegenerative disease.^56^ In this regard, in topics below we discussed the main pathologic events in separate sections but elaborating their interaction with other mechanisms involved in AD pathology.

The mTOR network comprises several crucial molecular targets that have been identified as^53^ potential therapeutic targets for various neurological disorders. Pharmacological manipulation of these targets within the mTOR network has shown promise as a therapeutic approach. Several animal studies focusing on AD have demonstrated the beneficial effects of targeting the mTOR network, as summarized in Table 2. These studies provide valuable insights into the potential of mTOR‐related interventions in the treatment of AD.

**TABLE 2:** Effect of rapamycin in animal models of AD.

AD model	Intervention (route of delivery)	Treatment protocol	Key results	Ref.
Human amyloid precursor protein (PDAPP) mouse model	Chow supplemented with either encapsulated rapamycin (14 ppm)	For 10 months	Restored BBB integrity through upregulating specific tight junction proteins	[132]
Low‐density lipoprotei receptor‐null (LDLR_/_) mice modeling vascular cognitive impairment		Starting at 5–6 months of age	Downregulated matrix metalloproteinase‐9 activity	
PDAPP mouse model	Chow containing either microencapsulated rapamycin (2.24 mg/kg)	For 16 weeks	Increased chaperones levels which, in turn, maintained proteostasis	[51]
		Starting at 4 months of age		
PDAPP mouse model	Chow containing either microencapsulated rapamycin (2.24 mg/kg)	For 13 weeks	Prevented AD‐like cognitive deficits	[70]
		Starting at 4 months of age	Improved spatial learning and memory	
			Decreased Aβ42 levels	
			Increased autophagy	
Senescence accelerated mouse prone 8 (SAMP8)	Treatment of neurons taken from SAMP8 mice with either 0.5 or 1.0 μM rapamycin	Incubation for 3 days	Reduced the levels of phosphorylated tau and p70S6K (pT389) in SAMP8 mice	[80]
			Inhibited mTOR signaling	
			Promoted autophagy	
			Decreased the protein expression level of *Bcl‐2* in primary neurons	
APOE4 transgenic mice	Chow containing either microencapsulated rapamycin (2.24 mg/kg)	For 6 months	Restored cerebral blood flow, BBB integrity, and glucose metabolism	[133]
			Restored brain vascular functions and metabolic functions	
			Attenuated spatial learning deficits	
			No change in anxiety level	
3xTg‐AD mice	Chow containing either microencapsulated rapamycin (2.24 mg/kg)	For 10 weeks	Reduced Aβ42 levels and deposition	[67]
			Rescued cognitive deficits	
			Ameliorated Aβ and tau pathology by increasing autophagy	
PDAPP mouse model	Chow containing either microencapsulated rapamycin (2.24 mg/kg)	For 16 weeks	Improved spatial learning and memory	[131]
		Starting at 7 months of age	Increased vascular density	
			Reduced cerebrovascular amyloid angiopathy and Aß Plaques	
			Induced nitric oxide (NO)‐dependent vasodilation	
			No change in glucose metabolism was observed	
Ts65Dn mouse model of Down syndrome	Intranasal rapamycin dose of 1 μg/mouse	For 12 weeks	Improved cognitive performances	[153]
		Starting at 6 months of age	Decreased mTOR hyper‐activation	
			Induced autophagy	
			Reduced aberrant APP levels and APP processing	
			Diminished tau hyper‐phosphorylation	
			Reduced oxidative stress markers	
Adeno‐associated viral vector‐based mouse model of early‐stage AD‐type tauopathy (AAV‐hTauP301)	Intraperitoneal injections of rapamycin at 15 mg/kg/day	3 times a week for either 3 or 5 weeks	Reduced mTOR activity and stimulated a marker of autophagic flux	[84]
			Protected against the tau‐induced neuronal loss, synaptotoxicity, reactive microgliosis, and astrogliosis	
			No changes in human tau mRNA or total protein levels were observed	
3xTg‐AD mice	Microencapsulated rapamycin was added at a concentration of 14 mg/kg to mouse chow 14 ppm	For 16 months, Starting at 2 months of age	Ameliorated learning and memory deficits	[71]
		For 3 months, Starting at 15 months of age	Reduced Aβ plaques and NFTs formation	
			Reduced microglia activation	
			Late rapamycin administration did not change AD‐like pathology and cognitive deficits	
P301S Tau transgenic mice	Intraperitoneal injection of rapamycin (15 mg/kg/week)	Twice weekly from the age of 3 weeks to 5.5 months of age	Reduced cortical tau tangles, tau hyper‐phosphorylation, and insoluble tau in the forebrain	[154]
			Decreased astrogliosis	
PDAPP mouse model			Restored neurovascular coupling and concomitantly reduced cortical amyloid‐beta levels	[155]
			Effectively treated memory deficits	
APP/PS1 mouse	Intraperitoneal injection of rapamycin (1 mg/kg)	For 2 months	Increased parkin‐mediated mitophagy and promoted fusion of mitophagosome and lysosome	[144]
		Starting at 4 months of age	Enhanced learning and memory viability, synaptic plasticity, and the expression of synapse‐related proteins	
			Decreased apoptosis and oxidative status	
			Recovered mitochondrial function	
3xTg‐AD mice	Intracerebroventricular injection of everolimus (0.167 μg/μL)	For 4 weeks	Reduced human APP/Aβ and human tau levels	[72]
		Starting at 4 months of age	Improved cognitive function and depressive‐like phenotype	

### 4.1 mTOR and amyloid‐beta

The amyloid hypothesis provides a solid framework to explain the Aβ mediated AD pathogenesis especially in the early disease process.^57^ The accumulation of Aβ may be evident 15–20 years before the clinical manifestation.^58^ Aβ aggregations, ranging from oligomers formation to fibrils deposition, are involved in AD progression including synaptic dysfunction, mitochondrial dysregulation, formation of NFTs, oxidative stress, glutamate excitotoxicity, calcium dysregulation, inflammatory responses, and finally neuronal loss.^57^, ^59^

The connection between mTOR and Aβ has been widely explored. Aβ plaques and both inhibition and activation of mTOR signaling counteract through known and unknown mechanisms.^60^ Research on transgenic mice models presents conflicting results. At the early stages of AD, activating mTOR signaling highly impacts Aβ production.^61^ On the other hand, an early report showed that phosphorylated forms of mTOR and p70S6k are decreased in the cortex of APP/PS1 transgenic mice compared to control. The authors also found that murine neuroblastoma cells treated with 20 μM Aβ1‐42 for 24 h exhibited reduced mTOR signaling.^62^ Caccamo et al.^63^ showed that in 3xTg mice brains, an animal model of AD, and cell lines stably transfected with mutant APP, the mTOR signaling and activity are significantly increased. Additionally, the authors observed that by genetically preventing the accumulation of Aβ, the mTOR activity levels in 3xTg mice could be close to the levels in wild‐type ones. Accordingly, intrahippocampal injections of an anti‐Aβ antibody normalized the mTOR signaling and decreased Aβ levels, suggesting that in 3xTg mice elevated levels of Aβ are essential to mTOR hyperactivity. Moreover, intrahippocampal injection of naturally secreted Aβ in the brains of wild‐type mice increased mTOR signaling.

To illustrate the underlying mechanisms, Aβ is known to interact with protein PRAS40, a component of the mTORC1, which directly binds to mTOR and decreases the mTOR activity.^64^ Although the mechanisms remain poorly understood, research shows that Aβ upregulates mTOR activity by phosphorylating PRAS40, since blocking the phosphorylation of PRAS40 can prevent the Aβ‐mediated mTOR activity upregulation.^63^ Accordingly, the brains of AD patients and 3xTg‐AD mice display significantly higher phosphorylated PRAS40.^65^ PRAS40 phosphorylation, in turn, is regulated by Pim 1, a protein kinase of the proto‐oncogene family. In this context, administering Pim1i, a selective Pim1 inhibitor, in 3xTg‐AD mice yielded interesting results. The Pim1i‐treated 3xTg‐AD mice exhibited better performance in spatial reference and working memory than their non‐transgenic and vehicle‐treated counterparts through declines in phosphorylated PRAS40 levels as well as Aβ reduction.^65^

Previous research has provided conflicting findings regarding the relationship between mTOR signaling and AD. Hyper‐activated mTOR signaling was initially reported in 9‐month‐old APP/PS1 mice^66^; however, later research found lower levels of mTOR signaling in 12‐month‐old APP/PS1 mice compared to age‐matched wild‐type mice.^62^ Similarly, in 3xTg‐AD mice, the increase in mTOR signaling was found to depend on both age and brain region.^63^, ^67^ Post‐mortem studies on AD brains consistently demonstrated upregulated mTOR signaling, which aligned with the results observed in 3xTg‐AD models.^63^, ^68^ Confocal microscopy data showed a direct interaction between mTOR signaling and Aβ42 (a hallmark protein in AD) within neurons.^69^ The co‐localization of Aβ42 with mTOR and p70S6K (a downstream effector of mTOR) in neurites of APP transgenic mice suggested that components of the mTOR signaling pathway are regulated by intraneuronal Aβ.^69^ While there is evidence supporting a direct interaction between mTOR and Aβ, it is important to note that the concept is complex, and in vitro and in vivo studies often yield conflicting results. However, the mTOR pathway can be considered one of the pathways through which Aβ toxicity is exerted, supporting the notion that reducing mTOR signaling in AD may be an effective treatment strategy. In line with this, a study by Spilman et al.^70^ demonstrated that chronic treatment with rapamycin, an mTOR inhibitor, for 13 weeks could reduce Aβ42 levels in the brains of a transgenic mouse model of AD. Additionally, in a study using the 3xTg‐AD mice, early administration of microencapsulated rapamycin was found to reduce amyloid plaque load and levels of soluble and insoluble Aβ40 and Aβ42, while late administration had less effect.^71^ Intracerebroventricular infusion of everolimus, an mTORC1 inhibitor, also showed reduced levels of APP/Aβ and tau in brain regions of symptomatic 3xTg‐AD mice.^72^

### 4.2 mTOR and tau

Tau is a microtubule‐associated protein and is mainly based on axons to ensure neural connections and microtubule stability. However, when hyper‐phosphorylated, its functions are impaired. The production of toxic tau oligomers, and further NFTs, also known as tauopathies, will play a key role in AD pathogenesis.^73^ The evidence linking tau and mTOR is less controversial. In general, mTOR‐related pathways are involved in the formation, hyper‐phosphorylation, degradation, and aggregation of NFTs.^74^ It is thought that the upregulation of the mTOR pathway can increase the level of hyper‐phosphorylated tau. To illustrate, mTOR activation impairs autophagy, a mechanism which reduces the accumulation of Aβ. When autophagy is impaired, the higher levels of piled‐up Aβ stimulate hyper‐phosphorylation of tau and the formation of NFTs.^75^ Various kinases phosphorylate tau including cAMP‐dependent protein kinase, Cyclin‐dependent protein kinase 5 (Cdk5), stress‐activated protein kinases (SAPK), AMPK, GSK3, mitogen‐activated protein kinases, and Protein Kinase A. According to a great body of research, AMPK activation precedes tauopathy, suggesting its involvement in hyper‐phosphorylation of tau and subsequent formation of NFTs.^76^ mTOR activity can drive tau accumulation in AD by increasing translation of tau mRNA via S6K1 activation as well as inhibiting protein phosphatase 2A (PP2A), as the major tau phosphatase.^77^ mTOR activity upregulation increases cytosolic tau, intracellular accumulation, and tau translocation to various cellular compartments, including endoplasmic reticulum, mitochondria, and Golgi apparatus, as seen in AD brains and human SH‐SY5Y neuroblastoma cells.^78^

In cellular and animal models, it has been shown that autophagy activators, for example, mTOR inhibitor drugs, can attenuate hyperphosphorylated tau and other aggregated misfolded proteins through stimulating the autophagy of NFTs and Aβ plaques.^79^ Wang et al.^80^ showed that upregulation of mTOR, phosphorylated 70S6 kinase (p70S6K) as mTOR substrate, and the protein expression levels of tau (pS396 or pS199) were reported elevated in hippocampus tissues isolated from senescence‐accelerated mouse prone 8 (SAMP8) newborn mice compared to control‐strain SAMR1 mice. Accordingly, administration of 0.5 μM rapamycin for 3 days dramatically decreased phosphorylated Tau protein (pS396 or pS199) expression levels as well as p70S6K in SAMP8 neurons in comparison with the untreated neurons‐SAMP8 group.^80^ The findings of the mentioned study suggested that the administration of rapamycin could ameliorate AD pathology by reducing the levels of p70S6K and phosphorylated tau. Interestingly, in contrast to this evidence, Hodges et al. proposed that mTOR could protect tau deposition. The authors aimed to find out how the levels of neuropathological proteins of AD were altered in the hippocampus of subset‐specific deletion of PTEN (NS‐PTEN) mice, manifesting with hyper‐activated mTOR activity. The authors observed reduced levels of GSK3α, GSK3β, tau, and APP in hippocampi of NS‐PTEN KO mice compared to wild‐type mice. Although mTOR inhibitor therapy could protect tau deposition, the authors note that a drastic reduction in mTOR activity could have a negative impact on the brain's memory and cognition as well.^8^

### 4.3 mTOR, gliosis, and inflammation

Glial cells, particularly activated microglia and reactive astrocytes, appear to play critical and interactive roles in AD pathogenesis. Neuroinflammation, including glia cell activation, is a remarkable characteristic of AD.^81^ For instance, elevated expression of inflammatory mediators has been reported in postmortem AD samples.^82^, ^83^ The level of Glial cell activation and their interplay are associated with the extent of brain atrophy and cognitive impairment; therefore, it might be expected that neuroinflammatory responses led by glial cells, and those of microglia and astrocyte in particular, exacerbate the neurodegeneration associated with AD.^81^ Rapamycin could putatively protect the entorhinal cortex and perforant pathway projection from reactive gliosis in a mouse model of early‐stage AD‐type tauopathy.^84^ Additionally, it is previously known that metformin has neuroprotective effects by upregulating fibroblastic growth factor 21 (Fgf21), leading to the AMPK/mTOR pathway activation and inhibition of tau phosphorylation inhibition. However, some research suggests that metformin may lead to cognitive impairments and enhanced gliosis.^85^

Neuro‐inflammation is considered one of the hallmarks in the brains of AD patients which is associated with inflammatory responses including enhancement in activated microglia and astrocytes, activated complement proteins, cytokines, and free radicals.^86^ Inflammation and oxidative stress promote initiation and progression of AD through facilitating Aβ production and NFTs formation which, in turn, contribute to worsening cognitive impairments.^87^

It has been revealed that rapamycin could exert neuroprotective effects via its anti‐inflammatory properties.^88^, ^89^ In this regard, chronic treatment with rapamycin could alleviate age‐dependent cognitive deficits in mice through downregulating interleukin‐1β (IL‐1β) and upregulating NMDA signaling in the hippocampus.^90^ Following lipopolysaccharide‐induced neuroinflammation response, rapamycin could suppress the expression of key proteins and cytokines including IL‐1β, IL‐6, hypoxia‐inducible factor‐1α (HIF‐1α), nuclear factor κB (NFκB) and modulate the neuronal inflammatory response in vitro and in vivo.^91^ The mTOR‐mediated phosphorylation of 4E‐BP1 and S6K1 pathways amplify in the hippocampus of AD rats. Conversely, suppressed mTOR signaling by rapamycin could increase pro‐inflammatory cytokines including IL‐6 and tumor necrosis factor‐α (TNF‐α) signaling pathways, as well as caspase‐3, and worsen learning performance in AD rats. Worthy to note that this discrepancy can be attributed to dosages of rapamycin, delivery route, animal species, and the range of age.^92^

### 4.4 mTOR and autophagy/apoptosis

Hyper‐ and hypoactivation of autophagy are involved in neurodegenerative diseases.^14^ AD is associated with neural death, which is an outcome or concomitant with Aβ and tau deposition.^93^

The relationship between the autophagy and the development of AD has garnered significant attention. In physiological conditions, autophagy operates consistently and effectively in normal neurons, but abnormal autophagy is involved in the pathogenesis of AD.^94^ The regulation of autophagy involves two pathways: mTOR‐dependent pathway and mTOR‐independent pathway. Nevertheless, both of these pathways were found to be abnormal in AD. It appears there is a bi‐directional relationship between AD pathology and autophagy.^95^ It has been established that autophagy induction can foster the clearance and degradation of Aβ and Tau in AD patients and animal models.^96^ On the other hand, as AD advances, autophagy becomes aberrant, and this process is accompanied by elevated Aβ and Tau levels which leads to impaired autophagy and mitophagy in AD.^97^, ^98^

mTORC1 regulates cell autophagy by recycling intracellular organelles through lysosomal degradation.^99^, ^100^ Accordingly, the involvement of mTOR signaling in AD pathogenesis is more implicated in the identified autophagy dysfunction in neurodegenerative diseases. Regarding the issue, the role of autophagy in Aβ pathogenesis should not be overlooked. Autophagy induces Aβ clearance and Aβ degradation. Moreover, autophagy is downregulated by mTOR activity; thus, dysregulation of autophagic processes is often expected in individuals with AD, which leads to facilitation of Aβ production and failure of its clearance.^61^, ^101^ mTORC1 activation reduces autophagy through phosphorylation of distinct substrates such as ULK1, which is involved in initiating the early steps of autophagy. Other substrates that mTORC1 phosphorylates include death‐associated protein 1 (DAP1), the nuclear translocation of the transcription factor EB (TFEB), and ATG13, all of which contribute to the inhibition of autophagy.^102^, ^103^ ULK1 phosphorylation by mTORC1 prevents AMPK from activating ULK1 in nutrient‐replete conditions.^104^ In various cellular conditions, the relative activity of mTORC1 and AMPK dictates the degree of autophagy. Moreover, phosphorylation and inhibition of TFEB by mTORC1 can partially control autophagy as TFEB controls the expression of genes engaged in lysosomal biogenesis.^105^, ^106^, ^107^ However, the exact pathways in which mTORC1 inhibits autophagy still remain ambiguous.

Over the past decade, the dysregulation of microRNAs (miRNAs) expression has been demonstrated in cellular, animal models, and even human AD subjects.^108^, ^109^ Several studies have shown that the expression of miR‐128 is dysregulated in AD brains.^110^, ^111^ In this respect, it has been shown that miR‐128 can promote autophagic flux by directly inhibiting mTOR, resulting in elevated Aβ clearance and reduced Aβ levels.^112^ Rapamycin binds to intracellular receptor, the FK506‐binding protein (FKBP12). The rapamycin‐FKBP12 forms an inhibitory complex that directly interacts with mTOR blocking its activity, which leads to autophagy induction.^113^ It has been shown that a novel small molecule named TH2849 from these derivative compounds has a significant binding connection with mTOR. TH2849 may induce autophagy most likely due to its suppression of the activation of mTOR, which subsequently dephosphorylates ULK1 and results in ULK1 complex formation.^114^

Consistently, mTOR hyperactivation can attenuate autophagy and apoptosis in individuals with AD, since mTOR is responsible for controlling cell growth and death by sensing environmental and nutritional status. Overactivation of PI3K/Akt/mTOR axis can downregulate autophagy, and functional autophagy protects neurons from apoptosis. Research on animal models shows that inhibition of mTOR pathway and its downstream signals by rapamycin may improve cognitive impairment by preventing neural apoptosis.^115^ Consistently, administration of Magnolol, an autophagy regulator compound, to transgenic mice model of AD inhibited apoptosis and decreased AD‐related pathologies by activating the AMPK/mTOR/ULK1 pathway.^116^ Moreover, autophagy induces Aβ clearance and Aβ degradation.^117^ Postmortem AD samples represent the alteration of mTOR signaling and autophagy at the early stages of AD. On the other hand, it seems that elevated Aβ levels are associated with reduced autophagy.^68^ Thus, dysregulation of autophagic processes is often expected in individuals with AD, which can result in facilitation of the process of Aβ production and failure of its clearance.^61^, ^101^

Given that apoptosis and autophagy are extremely linked together through various intrinsic and extrinsic pathways, alteration in apoptosis level is also crucial in the pathophysiology of AD.^118^ Caspase‐dependent apoptosis aligns with activating special pathways that bring about activation of specific proteases.^118^ Caspase 3 and apoptosis are activated when cultured hippocampal neurons are exposed to Aβ.^119^ Caspase 3, as a dominant caspase, contributes to amyloidogenic cleavage of the APP.^120^, ^121^ Moreover, caspase 3 can cleave pathologic soluble Tau hyper‐phosphorylated at Ser396 and Ser404^122^; thus, leading to NFTs formation. Similarly, Aβ‐induced caspase 3 activation can impair normal tau processing.^118^ Research on 3 transgenic AD mouse models, revealed that the anti‐apoptotic *Bcl‐2* gene resulted in the inhibition of caspase‐dependent cleavage of tau, inhibition of plaques and tangles formation, and improvement of memory.^123^ Paquet et al.^124^ conducted a clinical trial aiming to investigate the effect of previous Aβ immunization on the expression of various apoptotic proteins in post‐mortem human brain tissue. Thirteen cortexes of AD patients were immunized by administering AN1792 (iAD) and 27 cortexes of nonimmunized AD patients were immunolabeled to determine the pro‐apoptotic proteins engaged in AD. The results indicated that apoptosis is downregulated in residual neural and other cells.

Moreover, Bryostatin administration leads to the inhibition of apoptosis. Bryostatin also induces anti‐Aβ oligomers, anti‐hyper‐phosphorylated tau, and synaptogenesis. Farlow et al.^125^ conducted a double‐blind, placebo‐controlled, phase II study on 150 AD severe patients for 12 weeks to assess the safety, tolerability, and efficacy of Bryostatin in the treatment of moderate to severe AD. The result suggested that Bryostatin might be favor compared to placebo. To sum up, inhibiting the intrinsic apoptotic pathways may be beneficial to AD progression. However, more research is required to unravel the connection between apoptosis, anti‐apoptotic drugs, mTOR signaling, and AD physiopathology.

### 4.5 mTOR and vascular dysfunction

Vascular dysfunction can be the source of various pathogenic pathways that lead to neuronal damage, dementia, and AD development according to the vascular hypothesis of AD that was postulated in 1993.^126^ In vascular aging, disruption in endothelium‐dependent vasodilation chronically declines cerebral blood flow (CBF). The vascular dysfunction including cerebral amyloid angiopathy (CAA) and pathological alterations in vessel cell function, blood–brain barrier (BBB) permeability, aberrant immune cell recruitment, and direct vascular contribution to inflammation are related to neurodegenerative changes. On the other hand, these vascular impairments in turn take part in amyloid deposition, neurotoxicity, glial cell activation, and metabolic deficits in the AD brain.^127^ Consequently, the formation of feedback cycle and remarkable overlap between cerebrovascular dysfunction and AD leads to progressive exacerbation of neuronal and vascular pathology indicating synergistic/additive effects of both compartments on cognitive decline.^128^

mTOR signaling through specific mechanisms active in endothelial and smooth muscle cells of vasculature regulates vascular function acutely and chronically.^129^ Elevated mTOR signaling has been found in AD, and the mTOR network contributes to cerebrovascular dysfunction, subsequence CBF deficits, and cognitive decline.^130^ Chronic attenuation of mTOR pathway by rapamycin treatment could not only restore CBF and cerebrovascular density but also decrease CAA and micro‐hemorrhages, amyloid burden, and finally can block the progression of cognitive deficits in AD mouse brains through nitric oxide synthase (NOS) activation.^131^ Rapamycin or other rapalogs exerts protective effects on BBB integrity in transgenic APP mice and vascular cognitive impairment models through downregulating matrix metalloproteinase‐9 activity and upregulating tight junction proteins present in cellular connections.^132^ The ApoE 4 transgenic mice treated with rapamycin exhibited restoration in CBF, BBB integrity, and glucose metabolism compared to wild‐type controls in which preservation of vasculature and metabolism were associated with amelioration of learning deficits.^133^ Furthermore, mTOR is engaged in cerebrovascular and cognitive dysfunctions associated with atherosclerosis. In this context, rapamycin‐mediated inhibition of mTOR pathway could restore neurovascular functions and cardiovascular health in mice model of atherosclerosis.^134^ Available literature shows that rapamycin treatment of obese mice via inhibition of Akt/mTORC2 axis activity brings about vascular senescence suppression, endothelial sprouting, endothelial NOS activity, and vasodilation.^135^

Studies have shown that the APOE ε4 allele imposes a great risk for developing dementia, since it regulates several key genes which are involved in function of brain.^136^ A cohort study conducted by Robb et al.^137^ on 246 APOE ε4 carriers and non‐carriers aimed to test whether APOE ε4 status could impact cerebral oxygen homeostasis and cognitive performance. The authors found that APOE4 carriers are often manifested with lower cerebral oxygen metabolism, interacting with the pathogenesis of AD and related neurodegeneration. Additionally, the cognitive decline associated with AD may be caused by cerebral hypoperfusion,^138^ which is even more significant in APOE ε4 carriers.^139^ A longitudinal study was conducted on 950 APOE ε4 carriers and non‐carriers who were cognitively intact. Findings of the study suggested an interactive effect between and sex on cerebral perfusion trajectory during later pathological stages of AD.^140^ To further elaborate on the relation between APOE ε4, oxygen homoeostasis, and mTOR signaling, it is worthy to mention the research conducted by Lin et al. using multimodal in‐vivo imaging. The authors aimed to test if an early intervention using rapamycin could prevent the progression of AD‐like symptoms in pre‐symptomatic APOE ε4 transgenic mice by restoring neurometabolic and neurovascular functions. Compared to wild‐type controls, rapamycin‐treated mice showed improved cerebral blood flow and less disrupted blood‐barrier integrity accompanied by ameliorated learning deficits. The link mentioned above between mTOR signaling and abnormal brain metabolism and cerebral blood flow in AD, especially in APOE4 carriers, may add to the rationale of rapamycin clinical trials previously discussed in the paper.^133^

### 4.6 mTOR and synaptic plasticity

The synaptic impairment is an early event of AD and develops before neurodegeneration. The synaptic loss underlies the memory impairment in the initial phase of AD, and there is a solid correlation between the level of synaptic loss and dementia severity.^87^ Many evidence has indicated that prior to plaque formation, soluble oligomeric forms of Aβ lead to neurotoxicity at synapses, especially glutamatergic synaptic transmission which triggers synaptic failure and memory impairment.^129^, ^141^

The mTOR is involved in long‐lasting forms of memory formation and synaptic plasticity. mTOR is engaged in protein synthesis related to synaptic plasticity through 4E‐BP and S6K.^130^ Interestingly, mTOR signaling pathway is inhibited in hippocampal slices of AD transgenic mice as well as in hippocampal slices of wild‐type mice treated with Aβ1‐42, suggesting that mTOR dysregulation is correlated to synaptic plasticity impairment. In this regard, up‐regulation of mTOR pathway through pharmacological and genetic manners could restore long‐term potentiation and prevent Aβ‐induced impairment in synaptic plasticity.^69^ Pretreatment of cultured hippocampal neurons with rapamycin could inhibit BDNF‐induced synaptic plasticity and reduce late‐phase LTP expression induced by high‐frequency stimulation. Moreover, the localization pattern of several key components including mTOR, eIF‐4E, 4E‐BP1, and 4E‐BP2 proposes that these proteins are involved in coupling synaptic events with the protein translational machinery.^142^ However, mechanisms underlying the relationship between synaptic plasticity and mTOR signaling also remain controversial.

### 4.7 mTOR and learning/memory

Inhibiting mTOR signaling with rapamycin not only protects against Aβ and tau pathology but also may prevent memory impairments. Simen et al.^84^ evaluated the impact of the rapamycin, an mTOR inhibitor and autophagy stimulator, on the tau‐mediated neurodegeneration and synaptic loss in a mouse model of early‐stage AD‐type tauopathy. The results of the study displayed that intraperitoneal injection of rapamycin can alleviate neuronal, axonal, and synaptic loss in perforant pathway projection and the entorhinal cortex. The perforant starting from layer II of the entorhinal cortex ending in the hippocampal dentate gyrus has a central role in long‐term memory and is precisely delicate to damages caused by tauopathy.^84^, ^143^

In the study conducted by Wang et al.,^144^ it was observed that rapamycin not only improved cognitive deficits but also enhanced memory viability, learning, and the expression of synapse proteins in APP/PS1 mice. The authors suggested that cognitive function in the APP/PS1 group showed a direct correlation with autophagy activation and an inverse correlation with mTOR activity and Aβ plaque levels, while the levels of soluble or insoluble Aβ were not significantly associated with cognitive function. These results indicated a significant involvement of mTOR signaling in the cognitive performance of APP/PS1 mice. Similarly, Cassano et al.^72^ reported that the administration of everolimus, in their study led to improvements in cognitive functions such as novel object recognition, spatial memory, inhibitory passive avoidance, and depressive‐like phenotype in 3xTg‐AD mice.

On the other hand, given that balanced mTOR activity has a pivotal role in learning and memory, chronic inhibition of mTOR activity by rapamycin or other mTOR inhibitors may harm cognition in the long term.^145^ Sui et al.^146^ found that systemically administered rapamycin in mice can cause deficits in spatial memory retrieval but not acquisition.^147^ Moreover, when rapamycin was infused into the basolateral amygdala or dorsal hippocampus of the rat, novel object recognition became impaired.^148^, ^149^ The study on an 8‐month‐old APP/PS1 transgenic mice model for AD revealed that astaxanthin (Ast), as a carotenoid with potent antioxidant and neuroprotective properties, can activate the mTOR pathway and can ameliorate cognitive impairment, and suggests that the Ast may be beneficial for the treatment of cognitive impairments in AD. The authors suggest that Ast diminishes cognitive deficits of AD by enhancing the mTOR‐dependent mitochondrial dynamics, decreasing Aβ accumulation, and ameliorating synaptic damage.^150^ L‐3‐n‐Butylphthalide (L‐NBP), a compound extracted from the seeds of Chinese celery, can activate mTOR/AKT signaling and ameliorate learning and memory deficits following repeated cerebral ischemia–reperfusion injuries.^151^ In this regard, results from a study on rat models of vascular dementia (VD) revealed that the neuroprotective characteristics of NBP on learning/memory impairments are mainly derived through activation of the mTOR pathway which suppresses ischemia‐induced autophagy.^152^ Available data on the effects of mTOR inhibitor/activator drugs on learning/memory point to the critical involvement of mTOR signaling in learning and memory. However, much further work would be required to elucidate the exact association between learning/memory impairments in AD and mTOR signaling pathway.

## 5 THERAPEUTIC POTENTIAL

**FIGURE 1:**
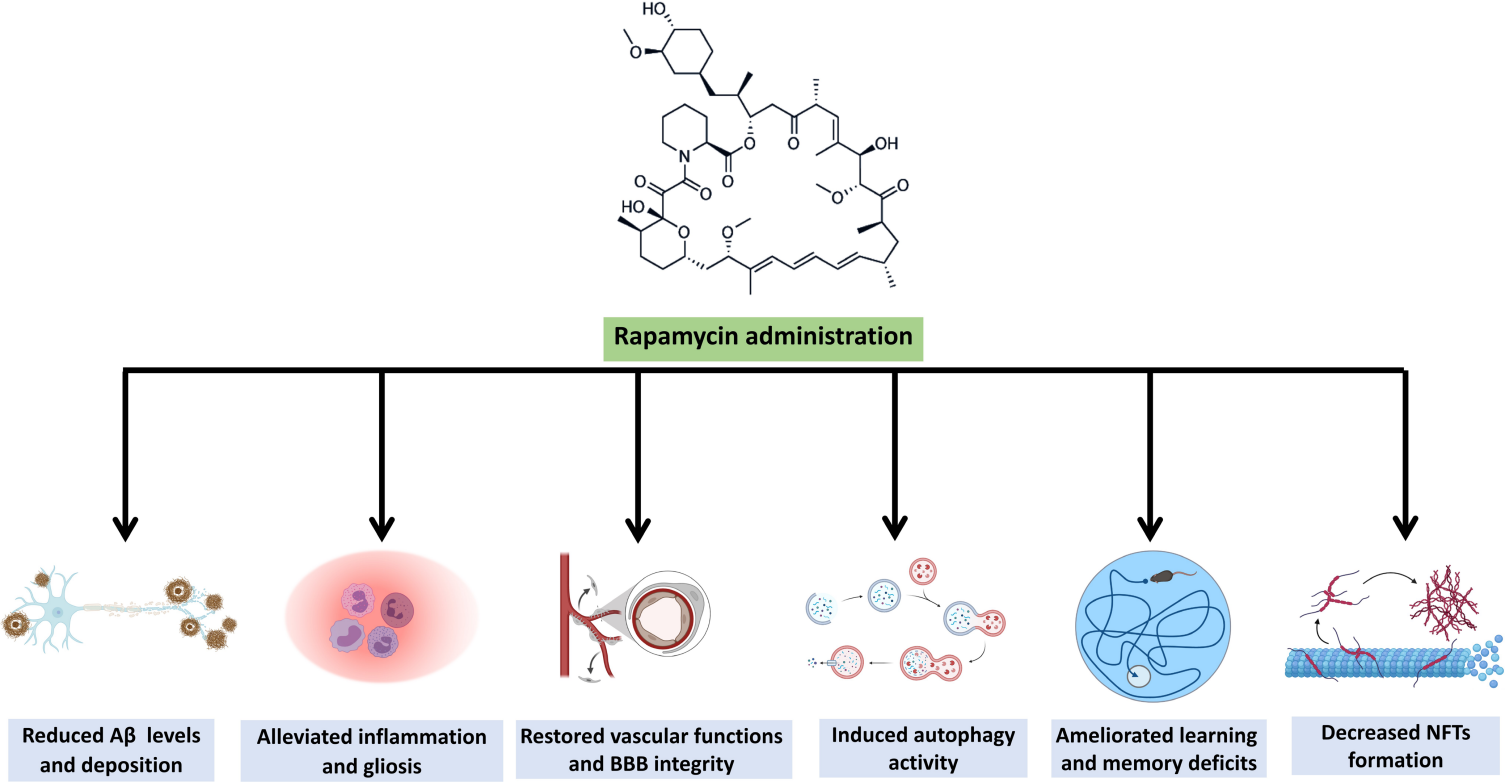
The therapeutic effects of rapamycin administration in Alzheimer's disease models.

In the past two decades, much research has indicated the massive complexity of the mTOR signaling network in mammalian brains.^156^ In general, hyperactivated mTOR signaling contributes to aging and AD progression. In this context, independent of AD pathology, rapamycin treatment may extend life span and delay aging per se, mediated through targeting of downstream signaling of mTORC1, enhancing autophagy, and reducing protein translation.^28^ As shown in Figure 1, administration of mTOR inhibitor drugs such as rapamycin may be beneficial in preventing the progression of AD, as mTOR signaling is involved in AD pathogenesis including the formation of NFTs and Aβ plaques.^75^, ^84^ Additionally, based on animal studies, administration of mTOR inhibitor drugs seems to be a potential pharmaceutical approach in patients with AD to ameliorate cognitive decline.^157^ Nonetheless, due to poor understanding of the underlying mechanisms, connections, and correlation between mTOR signaling and AD pathogenesis long‐term activation or inhibition of mTOR signaling might leave dire impacts.

The beneficial effects of rapamycin on AD pathology are paradoxical for synaptic plasticity and memory, and mTOR inhibition signaling might impair synaptic plasticity in the AD brain. As regards, balanced mTOR signaling is required for synaptic plasticity and memory, whereas chronic and complete inhibition of mTOR might exert deleterious effects on plasticity and long‐term memory.^69^ On the other hand, different concentrations of Aβ could result in differential effects on mTOR signaling.^145^ Shi et al.^158^ observed that in the *5XFAD* AD mouse model, mTOR activation was accompanied by decreased dendrite loss, enhanced Aβ clearance, and improved cognitive function. Extended administration of rapamycin even elicited further decrease of mTOR functioning and elevation of Aβ levels. This study contradicts previous studies that showed reducing mTORC1 could benefit the treatment of Alzheimer's disease. Although we believe that the controversial findings may stem from the different AD mouse models use, there may be multiple factors at play in the controversial findings. Low mTORC1 signaling is likely to be desirable in healthy state or early progression of the disease, while high levels are likely to be needed for brain health maintenance and microglial homeostasis at later stages.^159^ Thus, clinical risks of chronic treatment with mTOR inhibitors should be considered, and for translation into AD patients, it is essential to determine optimal dosage of rapamycin.^28^

It is important to note that the effects of rapamycin can vary between different animal models.^160^ A prophylactic dose of rapamycin significantly reduced plaques, tangles, and cognitive deficits in 2‐month‐old 3xTg‐AD mice. The induction of autophagy, however, had no effects on AD‐like pathology and cognitive deficits in 15‐month‐old 3xTg‐AD mice that had plaques and tangles.^71^ In the mouse hippocampus of APP/PS1, rapamycin helps promote Parkin‐mediated mitophagy. With rapamycin treatment, APP/PS1 mice showed enhanced recovery of cognitive function, synaptic plasticity, synapse‐related proteins, and immunity to cytochrome C‐mediated apoptosis.^144^ Rapamycin treatment in mouse model of early‐stage AD‐type tauopathy resulted in a reduction in mTOR kinase activity in the brain. This led to a decrease in phosphorylation of the mTOR substrate P70S6 kinase. Additionally, rapamycin inhibited the trans‐synaptic transfer of human tau expression to the dentate granule neuron targets, potentially preventing neurodegeneration and synapse loss in the perforant pathway.^84^ The complex nature of AD and the limitations of animal models necessitate further research to fully understand the potential benefits and risks of rapamycin in treating Alzheimer's disease in humans.

Although rapamycin is widely recognized as an inhibitor of mTOR, the off‐target effects of it should not be overlooked and the exploration of other targets of rapamycin will further elucidate its underlying mechanisms of action. Le Sun et al. demonstrated that STAT3 as a direct functional protein target of rapamycin. They showed that rapamycin, a macrolide compound with inhibitory function for mTOR, directly binds to STAT3 inhibiting its transcription factor activity. Furthermore, another transcription factor, c‐Myc, that up to now has been undruggable, is inhibited by rapamycin. Long‐term rapamycin treatment of xenograft mice results in a decrease of both STAT3 and c‐Myc expression. Therefore, rapamycin may serve as a lead compound for oncology drug development efforts targeting STAT3.^161^ In addition, geroprotective effects of chronic rapamycin treatment can be obtained with a brief pulse of the drug in early adulthood in female Drosophila and mice, without the adverse effects sometimes seen with chronic, long‐term dosing.^162^

## 6 CONCLUSIONS AND FUTURE PROSPECTS

Currently, there is no terminated clinical trial assessing the efficacy of mTOR inhibitor drugs in delaying or preventing the onset or progression of AD. The experimental studies have provided solid evidence for the contribution of mTOR signaling to AD pathogenesis. Upregulation of mTOR activity appears to be involved in the pathological cascade of AD, and mTOR inhibition or targeting its downstream substrates could supply attractive avenues for AD treatment. It has been also shown to be the most efficient pharmacologic compound directly attenuating the aging process and prolonging life span in animal models. Research on animal models shows that anti‐aging properties of rapamycin exceed increasing life span to inhibit age‐related dysfunctions or diseases such as cognitive decline and muscle loss.^12^ Additionally, treatment of different AD models with rapamycin led to improved autophagy and/or amyloidosis.^77^ However, the role of mTOR activation in AD seems complex.

Despite recent advances in understanding mTOR functioning, many questions regarding the application in the clinical setting remain to be answered. Furthermore, there are ongoing debates that rapamycin treatment may not be an efficient pharmacological approach in humans, since the efficacy of reducing the neuropathological hallmarks of AD is tested in the early phases of AD progression and not when the dire symptoms are present in patients. After the clinical diagnosis in humans, the lysosomal system is impaired, and treatment with rapamycin may even aggravate the impairment.^163^ Taken together, further efforts should be applied to optimize the therapeutic potential of mTOR inhibitors and minimize side effects. Furthermore, although beneficial effects of rapamycin have been proven in animal models of AD, it remains to be defined whether these results could be extended to AD patients as well. In this respect, determining the favorable dosing, timing, and treatment duration will be of dominant significance.

## AUTHOR CONTRIBUTIONS

**Samin Davoody** and **Afsaneh Asgari Taei**: Performed the literature review, wrote the first draft of the manuscript, designed the tables and figure, revised and edited the manuscript; **Pariya Khodabakhsh**: performed the literature review, revised and edited the manuscript; **Leila Dargahi**: overviewed the latest state of knowledge, revised and edited the manuscript, supervised the review process. All authors read and approved the final manuscript.

## FUNDING INFORMATION

None.

## CONFLICT OF INTEREST STATEMENT

All the authors declare that they have no conflicts of interest.

## CONSENT FOR PUBLICATION

All authors whose names appear on the submission approved the final manuscript and agreed to be published.

## Data Availability

Not applicable.
